# The Corticospinal Excitability Can Be Predicted by Spontaneous Electroencephalography Oscillations

**DOI:** 10.3389/fnins.2021.722231

**Published:** 2021-08-23

**Authors:** Guiyuan Cai, Manfeng Wu, Qian Ding, Tuo Lin, Wanqi Li, Yinghua Jing, Hongying Chen, Huiting Cai, Tifei Yuan, Guangqing Xu, Yue Lan

**Affiliations:** ^1^Department of Rehabilitation Medicine, The Second Affiliated Hospital of South China University of Technology, Guangzhou, China; ^2^Department of Rehabilitation Medicine, School of Medicine, South China University of Technology, Guangzhou, China; ^3^Department of Rehabilitation Medicine, Guangzhou First People’s Hospital, Guangzhou, China; ^4^Shanghai Key Laboratory of Psychotic Disorders, Shanghai Mental Health Center, Shanghai Jiao Tong University School of Medicine, Shanghai, China; ^5^Co-innovation Center of Neuroregeneration, Nantong University, Nantong, China; ^6^Translational Research Institute of Brain and Brain-Like Intelligence, Shanghai Fourth People’s Hospital Affiliated to Tongji University School of Medicine, Shanghai, China; ^7^Department of Rehabilitation Medicine, Guangdong Provincial People’s Hospital, Guangdong Academy of Medical Sciences, Guangzhou, China

**Keywords:** electroencephalography, transcranial magnetic stimulation, corticospinal excitability, network, power spectrum

## Abstract

Transcranial magnetic stimulation (TMS) has a wide range of clinical applications, and there is growing interest in neural oscillations and corticospinal excitability determined by TMS. Previous studies have shown that corticospinal excitability is influenced by fluctuations of brain oscillations in the sensorimotor region, but it is unclear whether brain network activity modulates corticospinal excitability. Here, we addressed this question by recording electroencephalography (EEG) and TMS measurements in 32 healthy individuals. The resting motor threshold (RMT) and active motor threshold (AMT) were determined as markers of corticospinal excitability. The least absolute shrinkage and selection operator (LASSO) was used to identify significant EEG metrics and then correlation analysis was performed. The analysis revealed that alpha2 power in the sensorimotor region was inversely correlated with RMT and AMT. Innovatively, graph theory was used to construct a brain network, and the relationship between the brain network and corticospinal excitability was explored. It was found that the global efficiency in the theta band was positively correlated with RMT. Additionally, the global efficiency in the alpha2 band was negatively correlated with RMT and AMT. These findings indicated that corticospinal excitability can be modulated by the power spectrum in sensorimotor regions and the global efficiency of functional networks. EEG network analysis can provide a useful supplement for studying the association between EEG oscillations and corticospinal excitability.

## Introduction

Transcranial Magnetic Stimulation (TMS) induces an electric field through a time-varying magnetic field, resulting in induced electric currents and changing the action potential of nerve cells in the cerebral cortex, thus affecting blood circulation, metabolism, and nerve excitability in the brain (Wanalee et al., 2015). As an effective non-invasive nerve stimulation technology, TMS is widely used in clinical practice and can be used to improve motor function in stroke patients, improve individual cognitive function, and reduce depression (Myczkowski et al., 2018; Chen et al., 2019).

Neuron oscillatory activity plays an important role in the cortical response to TMS. To clarify the interaction between neural activity and TMS, researchers explored the association between electroencephalography (EEG) and corticospinal excitability, defined as the cortical output in response to TMS. It was found that the fluctuation of EEG oscillations selectively affects corticospinal excitability (Berger et al., 2014; Bergmann et al., 2019; Ogata et al., 2019). Although EEG oscillations were assumed to regulate cortical excitability, the findings in previous studies were inconsistent. Some studies reported a correlation between corticospinal excitability and pre-stimulation alpha oscillation power (Sauseng et al., 2009) as well as beta oscillation power (Maki and Ilmoniemi, 2010; Hussain et al., 2019b) while others found no correlations between various EEG frequencies (Iscan et al., 2016). There are contradictions in these studies. Sauseng et al. (2009) found that the amplitude of motor evoked potentials (MEPs) is negatively correlated with the pre-stimulation alpha oscillation power, while Ogata et al. (2019) reported the opposite results. These conflicting results have evoked the need for further investigation of the relationship between EEG oscillations and corticospinal excitability to obtain reliable results.

Interestingly, previous studies focused on how EEG oscillations in sensorimotor regions modulated corticospinal excitability, while ignoring the effects of brain global network activity. Early neuroscience research focused on the function of single brain regions, while modern approaches tend to use complex network methods to analyze the structure and dynamic behavior of neural networks. Brain regions such as the frontal and parietal lobes regulate corticospinal excitability (Cattaneo and Barchiesi, 2011). A previous study showed that corticospinal excitability was regulated by the attention network, providing another perspective for understanding the association between corticospinal excitability and brain networks (Avenanti et al., 2018). A recent study indicated that TMS efficacy was modulated by the functional state of the target brain network (Schiena et al., 2020), suggesting that researchers should pay more attention to the impact of brain networks on corticospinal excitability rather than a single brain region.

In the field of brain networks, techniques for the construction and analysis of brain networks are still evolving. As a branch of scientific computing, graph theory involves the construction of a network by defining a series of nodes and connecting edges. This model fits the pattern of brain activity, which makes it a great tool for brain functional segmentation and integration (Sporns, 2018). The global efficiency and the clustering coefficient can be calculated using graph theory to measure the features of the brain network. Graph theory analysis of EEG has been gradually applied to describe neural electrophysiological activity (Rubinov and Sporns, 2010). A previous study showed that adjusting brain excitability through transcranial direct current stimulation can change the small-world propensity in brain networks (Vecchio et al., 2018), suggesting that graph theory can be a useful approach to study the interactions between brain networks and neural regulation technology.

For the purpose of this study, we aimed to explore the association between corticospinal excitability and EEG oscillations. The resting motor threshold (RMT) and active motor threshold (AMT) were identified as markers of corticospinal excitability according to previous study (Stefanou et al., 2020). Innovatively, we used graph theory analysis methods to construct brain networks to explore the relationship between the network properties and corticospinal excitability. Considering the factors mentioned above, we assumed that corticospinal excitability depends not only on the neural activity of the motor region, but also on the functional activity of the brain network. Clarifying the mechanism by which oscillating brain activity modulates corticospinal excitability will help elucidate how TMS works, which is conducive to enhancing the effect of TMS by stimulating the brain based on the current neural state.

## Materials and Methods

### Participants

A total of 32 right-handed individuals (mean age: 21.53 ± 1.46; 7 men), all college students, were included in this study. The participants did not take psychotropic drugs and had no history of central nervous system diseases or head trauma. Individuals with contraindications to TMS were excluded (Rossi et al., 2021). Before the experiment, the participants were required to get enough sleep to maintain a good mental state during the experiment. All participants were informed of the purpose and content of the experiment and signed informed consent forms before the experiment. This study was approved by the Ethics Committee of Guangzhou First People’s Hospital.

### EEG Acquisition and Processing

#### EEG Acquisition

Resting-state EEG were recorded before application of TMS. The EEG data collection was performed in the electrophysiological laboratory and the room was quiet during EEG recording. The participants sat in a comfortable chair and their resting EEG was recorded for 10 min while their eyes were closed. They were required to remain awake throughout the recording. EEG data were recorded by using 128-channel HydroCel Geodesic Sensor Net (Electrical Geodesics, Inc., Eugene, OR, United States), and the Cz electrodes were used as the online reference. The impedances of all electrodes were kept below 10 kΩ by input impedance amplifiers (Geodesic EEG system 400). The signal was amplified at a sampling rate of 2,048 Hz and filtered through a 0.1–100 Hz band-pass filter. Data were processed offline after continuous EEG acquisition.

#### EEG Processing

*E*lectroencephalography processing was conducted with Matlab R2013a (The MathWorks, Natick, M*A*, United States) and eeglab12.0.^1^ After reducing the sampling rate to 1,000 Hz, the data were filtered through 0.1–40 Hz with a finite impulse response (FIR) filter. Continuous data were segmented into 2 s for each epoch. Bad data were defined when the amplitude exceeded ± 100 μV. Independent components analysis (ICA) was conducted to eliminate electro-oculograms after removing bad epochs and interpolating electrodes with high noise.

To obtain spectrum information for the EEG data, Fast Fourier Transform (FFT) was used to decompose the data. We calculated the power of the four frequency bands: delta (1–4 Hz), theta (4–8 Hz), alpha1 (8–10 Hz), alpha2 (10–13 Hz), beta1 (13–20 Hz), and beta2 (20–30 Hz). In accordance with previous research, data collected from the cluster of EEG electrodes around C4 were averaged to represent the activity of the sensorimotor region (Bayram et al., 2015).

As for functional connectivity, the phase lag index (PLI) was applied to characterize the connections between the electrode pairs because it can eliminate the volumetric conduction effect. Band-pass filtering was performed on the electrode signal, and Hilbert Transform was performed on the filtered electrical signal to extract the phase at each time point. Afterward, the PLI of each electrode pair in the six frequency bands was calculated. PLI was calculated using the following formula (Su et al., 2017).

(1) $$\mathrm{PLI}=\left|\left\langle \mathrm{sign}\left[\sin\left(△\varphi \left(t_{k}\right)\right)\right]\right\rangle \right|$$

The range of the PLI value was 0–1; 0 indicated that the two signals did not have a linear dependence in this frequency band, while 1 indicated complete synchronization.

In this study, the GRETNA toolbox^2^ was used for graph theoretical network analysis (Wang et al., 2015). The undirected weighted network was set up using electrical poles as nodes and the PLI value as the edge weight (de Waal et al., 2014). Based on previous studies, the clustering coefficient and efficiency, which are the most commonly used metrics, were selected to characterize the complex networks (Zomorrodi et al., 2019). Since there was no definite method for selecting a single threshold, referring to previous studies, we integrated the metrics over the entire threshold range to obtain the area under the curve (AUC) to characterize the brain network (Wang et al., 2015; Yan et al., 2017).

The clustering coefficient is defined as the ratio of the actual number of edges between a given node and its neighbors and the total number of possible edges between these nodes, and is used to measure the tightness between a node and its neighbor nodes in a network.

(2) $$C_{i}=\frac{2l_{i}}{k_{i}\left(k_{i}-1\right)}$$

The clustering coefficient of the whole network is the average of all nodes in the network.

(3) $$C_{global}=\frac{1}{n}\sum_{i\in N}C_{i}$$

Efficiency refers to the reciprocal of the harmonic average distance between all nodes in the network. The efficiency of a node is used to measure the information transmission capacity of the given node in the network.

(4) $$E_{i}=\frac{\sum_{j\in N,j\neq i}{\left(d_{ij}\right)}^{-1}}{n-1}$$

The global efficiency is the average of all nodes in the network.

(5) $$E_{global}=\frac{1}{n}\sum_{i\in N}E_{i}$$

For the formula and interpretation of these metrics, see Rubinov and Sporns (2010). The clustering coefficient and node efficiency of the motor cortex are the average values from the electrodes in the motor region.

### TMS Procedure

Resting motor threshold and AMT was recorded immediately after EEG acquisition. Stimulation was applied using a figure-eight coil connected to the NS5000 Magnetic Stimulator (YIRUIDE Medical Co., Wuhan, China) with a maximum magnetic field intensity of 2.5 T. The participants assumed a sitting position with the body relaxed. The coil was placed in the projection of the primary motor cortex on the body surface of the right hemisphere, tangent to the scalp with the handle points facing backward and 45° away from the midline. This orientation induced a posterolateral to anteromedial current in the brain that preferentially activated the cortical-spinal system through horizontal cortical–cortical connections (Premoli et al., 2014). A single TMS pulse was applied to the M1 region, and MEPs from the left first digital interosseous (FDI) muscle were recorded with surface electromyography. The resting motion threshold was determined using the relative frequency method, defined as the minimum intensity that was sufficient for the MEPs to reach an amplitude > 50 μV in at least five out of ten of the subsequent stimuli. AMT was determined during muscle contraction (approximately 20% of maximum muscle strength) and was defined as the minimum intensity able to elicit MEPs (peak amplitude > 200 μV) in 50% of the subsequent stimuli. Furthermore, we used the neuro-navigation system (Visor2, ANT Neuro, Hengelo, Netherlands) to record the FDI hot spots to ensure that the coil would not deviate from the stimulus target throughout the experiment.

### Statistical Analysis

To confirm the relationship between spontaneous EEG oscillations and corticospinal excitability, the least absolute shrinkage and selection operator (LASSO) was used to identify significant features and correlation analysis was conducted for these features. The basic idea of LASSO is that it compresses the coefficient of variables by adding penalty functions to the model and eliminates variables with a regression coefficient of 0 to facilitate the selection of variables (Li et al., 2017). The R 4.0.5 software^3^ and glmnet package (Friedman et al., 2010) were used to perform LASSO. The parameter Lambda (λ) was tuned by 10-fold cross-validation based on the minimum criteria. To obtain the best fitting effect, the model with minimum λ was chosen. Correlations between significant features extracted by LASSO and corticospinal excitability were then evaluated. Statistical analysis was conducted using SPSS 25.0 (SPSS Inc., Chicago, IL, United States). The Shapiro-Wilk normality test was performed on all variables, and Pearson correlation analysis was used for variables with normal distribution; otherwise, Spearman correlation analysis was applied. *p* < 0.05 was considered statistically significant.

## Results

### Descriptive Results

The range of RMT was from 21 to 73% MSO and the mean value was 46.25% MSO [standard deviation (SD) = 13.579]. AMT ranged from 12 to 57% MSO and the mean value was 32.31% MSO (SD = 11.183). The descriptive results for other variables are shown in Table 1.

**TABLE 1:** Descriptive results of electroencephalography (EEG) metrics.

	Delta	Theta	Alpha1	Alpha2	Beta1	Beta2
Power spectrum	2.062 ± 1.030	1.247 ± 0.493	1.412 ± 0.672	1.359 ± 0.697	0.736 ± 0.338	0.497 ± 0.193
**Global metric**						
CC	0.163 ± 0.016	0.160 ± 0.017	0.140 ± 0.023	0.143 ± 0.026	0.156 ± 0.028	0.136 ± 0.028
Efficiency	0.089 ± 0.003	0.079 ± 0.002	0.077 ± 0.008	0.076 ± 0.012	0.053 ± 0.004	0.045 ± 0.003
**Nodal metric**						
CC	0.165 ± 0.030	0.161 ± 0.026	0.130 ± 0.050	0.133 ± 0.056	0.170 ± 0.043	0.143 ± 0.037
Efficiency	0.084 ± 0.007	0.080 ± 0.007	0.072 ± 0.024	0.070 ± 0.022	0.053 ± 0.007	0.048 ± 0.006

*Data are presented as mean ± standard deviation. CC, clustering coefficient.*

### Extraction of Features

**FIGURE 1:**
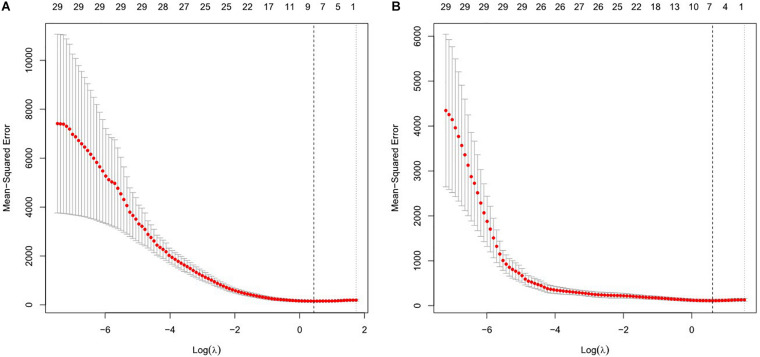
Features selection using the least absolute shrinkage and selection operator (LASSO) regression model for resting motor threshold (RMT) **(A)** and active motor threshold (AMT) **(B)**. The horizontal axis plotted value of log λ and the vertical axis plotted mean squared error. The dotted vertical line was plotted at the optimal λ values based on minimum criteria.

Least absolute shrinkage and selection operator was used to construct the regression model for EEG parameters to predict RMT, and the regression model with the minimum λ value was selected because it had the best prediction. The minimum λ was 1.548, and eight potential predictors were identified, including alpha2 and beta1 oscillations power in the sensorimotor region, nodal efficiency in the alpha1 and alpha2 bands, global efficiency in the delta, theta, and alpha2 bands, and global clustering coefficient in the theta band. In the model used to predict AMT, the minimum λ was 1.844. Alpha2 oscillations power in the sensorimotor region, nodal efficiency in the theta and alpha1 bands, global efficiency in the theta and alpha2 bands, and global clustering coefficient in the beta2 band were selected as predictors. All significant factors were included in the subsequent correlation analysis. See Figure 1.

### Correlation Analysis

**FIGURE 2:**
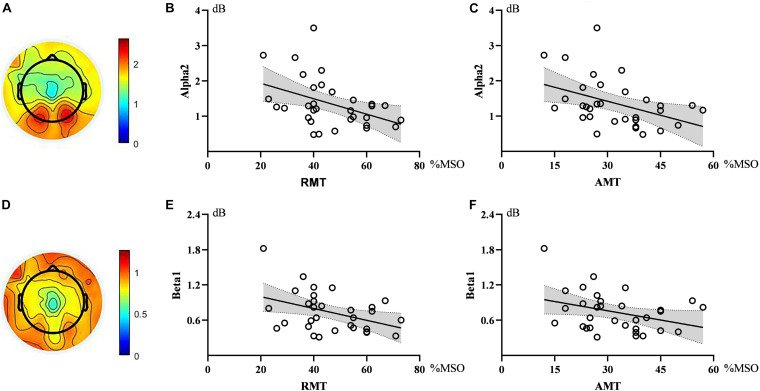
Topographic maps and scatter diagrams. Topographic maps showed the power of alpha2 oscillations and beta1 oscillations. The color bar represents the value of power **(A,D)**. Scatter diagrams showed the correlation between electroencephalography (EEG) oscillations and motor threshold **(B,C,E,F)**. There were significant negative correlations between alpha2 and RMT as well as AMT.

Correlation analysis showed that the power of alpha2 oscillations in the sensorimotor region was negatively correlated with RMT (ρ = −0.376, *p* = 0.034). Similarly, alpha2 power in the sensorimotor region showed an inverse correlation with AMT (ρ = −0.432, *p* = 0.014). The strength of the correlation between beta1 and RMT did not reach a statistically significant level (ρ = −0.328, *p* = 0.066), similar to that between beta1 and AMT (ρ = −0.314, *p* = 0.080). See Figure 2.

**FIGURE 3:**
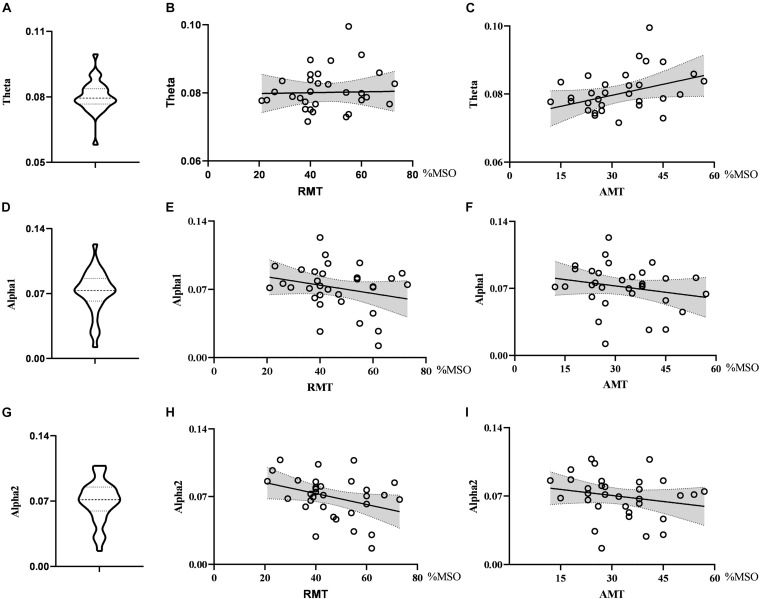
Violin diagrams and scatter diagrams. Violin diagrams showed the distribution of nodal efficiency in theta, alpha1, and alpha2 band **(A,D,G)**. Scatter diagrams showed the correlation between nodal efficiency and motor threshold **(B,C,E,F,H,I)**.

When exploring the relationship between the nodal metrics and RMT, we found that RMT was negatively correlated with nodal efficiency in the alpha2 band, but the correlation did not reach statistical significance (*r* = −0.347, *p* = 0.051). Nodal efficiency in the theta band had no correlation with RMT (*r* = 0.023, *p* = 0.900) and AMT (*r* = 0.336, *p* = 0.060) and there was no correlation between nodal efficiency in the alpha1 band and RMT (*r* = −0.247, *p* = 0.173) or AMT (*r* = −0.210, *p* = 0.249). See Figure 3.

**FIGURE 4:**
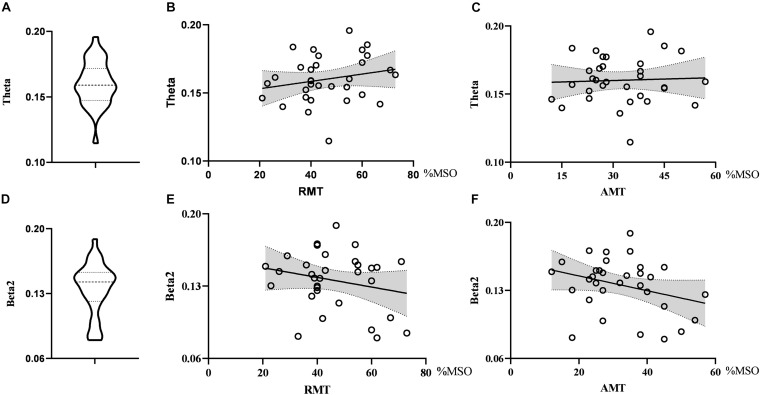
Violin diagrams and scatter diagrams. Violin diagrams showed the distribution of clustering coefficient in theta and beta2 band **(A,D)**. Scatter diagrams showed the correlation between clustering coefficient and motor threshold **(B,C,E,F)**.

**FIGURE 5:**
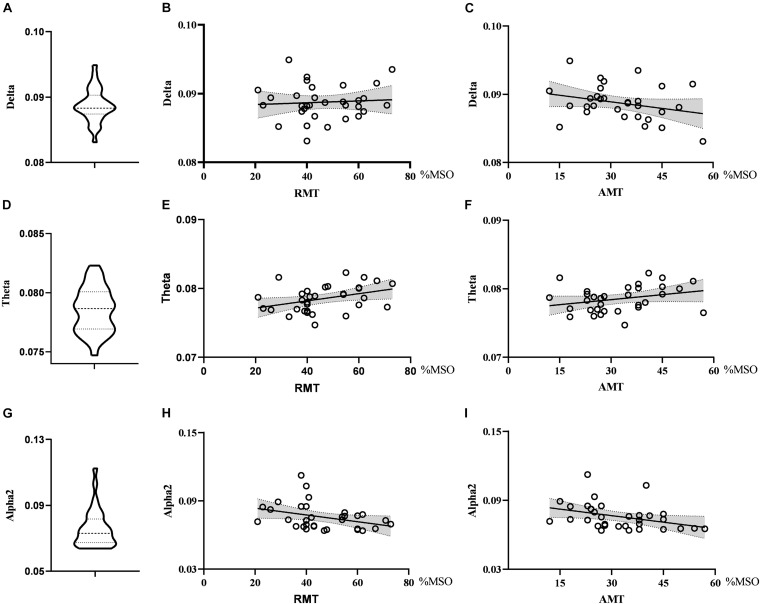
Violin diagrams and scatter diagrams. Violin diagrams showed the distribution of global efficiency in delta, theta, and alpha2 band **(A,D,G)**. Scatter diagrams showed the correlation between global efficiency and motor threshold **(B,C,E,F,H,I)**. There was significant positive correlation between global efficiency in theta and RMT. And there were significant negative correlations between global efficiency in alpha2 and RMT as well as AMT.

As for global metrics, we found that the global efficiency in the theta band was positively correlated with RMT (*r* = 0.374, *p* = 0.035), while the global efficiency in the alpha2 band was negatively correlated with RMT (ρ = −0.363, *p* = 0.041), showing the opposite trend. Similarly, the global efficiency in the alpha2 band was negatively correlated with the AMT (ρ = −0.427, *p* = 0.015) and the correlation strength between the global efficiency in the theta band and AMT did not reach the statistically significant level (*r* = 0.291, *p* = 0.106). There was no correlation between global efficiency in the delta band and RMT (*r* = 0.074, *p* = 0.688) as well as AMT (*r* = −0.281, *p* = 0.119). Further, the global clustering coefficient in the theta and beta2 band had no significant correlation with RMT or AMT. See Figures 4, 5.

## Discussion

In this study, we explored the relationship between EEG oscillations and corticospinal excitability. Analysis revealed that the alpha2 power in the sensorimotor region showed an inverse correlation with RMT and AMT. Innovatively, we explored the relationship between brain activity and corticospinal excitability from the perspective of the functional network and found that the global efficiency in the theta band was positively correlated with RMT. Additionally, the global efficiency in the alpha2 band was negatively correlated with RMT and AMT. These findings indicated that the power spectrum in sensorimotor regions and the global efficiency of functional networks modulate corticospinal excitability, which provides an important basis for understanding the interactions between neural electrical activity and TMS.

### The Correlation Between Power Spectrum in Sensorimotor Regions and Corticospinal Excitability

The spontaneous oscillation of EEG reflects the rhythmic changes in the membrane potential of neurons and thus reflects the current excitatory–inhibitory balance of underlying neuronal cell assemblies (Klimesch et al., 2007; Jensen and Mazaheri, 2010; Schulz et al., 2014). Each EEG band is associated with different cognitive and behavioral functions. Low-frequency EEG oscillations are associated with responsible motivation, emotion, and reward processing, while high-frequency EEG oscillations may reflect cognitive processes such as attention control, memory encoding, and recognition (MacLean et al., 2012; Sandler et al., 2016; De Pascalis et al., 2020). Alpha oscillations are the most significant phenomenon in human EEG recordings, and their occurrence and function are some of the basic research topics in neuroscience. At first, researchers believed that the alpha activity reflected the idle state of the brain, but the current leading interpretation of alpha oscillations is that alpha oscillations causally determine excitability and modulate signal processing instead of passively respond to stimulus. Converging evidence suggests that alpha oscillations are related to the cyclic regulation of neuronal excitability and can affect the response of neurons to sensory stimuli (Palva and Palva, 2011). Currently, researchers recognize that the alpha oscillations contain at least two sub-components. The first is lower alpha or alpha1, which is endogenous and independent of any internal or external stimuli. The second is upper alpha or alpha2, mainly exists in the sensorimotor cortex, and is related to the function of the sensorimotor system (Vecchio et al., 2018).

In this study, RMT and AMT decreased when the alpha2 oscillation power increased, indicating an inverse correlation between alpha2 oscillations and corticospinal excitability. A number of studies support the view that the alpha rhythm reflects cortical excitability. Previous studies have shown that central alpha oscillations (mu rhythms) are associated with the resting state of the primary sensory and motor cortex by correlating the rhythm strength with fMRI blood signals (Ritter et al., 2009). Alpha oscillations in the central region are suppressed during movement and return to the baseline when the movement ends (Hao et al., 2019). According to the pulsed inhibition hypothesis, alpha oscillations are associated with the underlying localized global suppression of neuronal activity in cortical circuits, with high alpha power representing the suppressed state and low alpha power representing the excited state (Palva and Palva, 2011; Schulz et al., 2014). Since RMT and AMT are inversely associated with cortical excitability, similar to alpha oscillations, there should be a positive correlation between RMT, AMT, and alpha oscillations. However, in this study, alpha2 oscillations were negatively correlated with corticospinal excitability, which seems controversial. A recent study explored the relationship between pre-stimulus EEG oscillations and MEPs, and the results showed that high alpha and low beta power before stimulation could lead to high MEP amplitudes (Ogata et al., 2019). In a real-time TMS-EEG study, researchers explored the relationship between the phase of the alpha oscillations in the sensory motor area and MEPs. They found that the MEP amplitude increased during the high alpha power, indicating that the alpha oscillations could promote corticospinal excitability (Bergmann et al., 2019). These results were consistent with our findings. The discrepancies between studies may be caused by different experimental designs. Previous studies proved the negative correlation between alpha oscillations and cortical excitability mainly by exploring the changes of alpha rhythm during motor tasks, such as voluntary movement and motor imagery, whereas our study recorded EEG and motor threshold at rest and the task state would contribute to the relationship between EEG oscillations and corticospinal excitability. Our results suggested that alpha oscillations can modulate the cortical response to TMS.

The relationship between beta oscillations and cortical excitability has been studied using different modalities. Generally, beta oscillations in the motor cortex decrease in amplitude during movement and increase in amplitude when movement stops (Darch et al., 2020). Hussain et al. (2019a) found that the beta rhythm power before TMS stimulation can be used to predict the amplitude of MEPs. The stronger the beta rhythm power is, the larger the MEP amplitude will be, indicating that the beta rhythm is positively correlated with corticospinal excitability. These findings may be because beta activity reflects the activity of the II/III interlayer neurons, which could regulate the excitability of the spinal cord by activating descending corticospinal neurons (Hussain et al., 2019b). In this study, beta1 oscillation was a significant feature in the regression model, and correlation analysis showed no significant correlation between beta oscillations and corticospinal excitability. This may be due to the small sample size. Nevertheless, the results of this study are valuable, because previous studies focused on how alpha oscillations regulate corticospinal excitability and beta oscillations were used to be ignored. Future studies can further explore the relationship between beta oscillations and corticospinal excitability.

### The Correlation Between Efficiency of Network and Corticospinal Excitability

With the advancement of neuroscience, the network connection pattern formed by the cooperation of multiple brain regions has been identified as the physiological foundation for information processing in the brain. The small-world network is the most commonly studied complex network which has both a large clustering coefficient and a small path length (Bassett and Bullmore, 2006). It examines the brain’s functional connectivity architecture, focusing on the brain’s ability to integrate and transmit information between different regions. Efficiency is an important metric to measure the ability of network information exchange, and its contribution to cortical excitability has attracted the attention of researchers. Blain-Moraes et al. (2017) investigated the neural activity of subjects under general anesthesia and found that while the subjects were unconscious, the alpha network efficiency decreased and the alpha network clustering coefficient increased significantly. This showed that the efficiency of the alpha network is positively correlated with cortical excitability. Previous studies have shown that the efficiency of the alpha network increased during exercise, possibly due to increased metabolism and cortical arousal during exercise (Tamburro et al., 2020). High global efficiency corresponds to fast information transmission. Our study showed that RMT decreased with the elevation of alpha network efficiency, indicating that brain activity increased with the enhancement of the alpha network information processing capacity, making it easier to respond to TMS.

Theta oscillations are often considered to be related to cognitive functions such as memory and emotion (Wang et al., 2018), and a few studies show that the theta rhythm contributes to the movement process. Popovych et al. (2016) reported that a significant phase-locking effect in the delta-theta frequency band could be observed in the M1 area when individuals completed an exercise task, suggesting that the theta oscillations were related to movement and indirectly showing that the theta rhythm contributes to the excitability of the motor cortex. Storti et al. (2016) used graph theory to analyze the brain network characteristics of individuals during movement and found that the betweenness in the theta band of the motor cortex increased significantly, providing a new perspective for exploring the theta network and the excitability of the motor cortex. Interestingly, in this study, the modulating effects of the global efficiency of the theta band and alpha band on RMT were in the opposite direction, which may indicate that different rhythm networks regulate cortical excitability in different directions and coordinate with each other to regulate brain activities.

In this study, the LASSO regression showed that the clustering coefficient in the theta and beta2 bands was the predictor of the motion threshold, but the correlation analysis results indicated that there was no correlation. This may result from the differences of statistical methods. Correlation analysis is univariate analysis, while LASSO is a multivariate analysis method and the results would be affected by the interaction between variables. The combination of clustering coefficients and other parameters can provide more comprehensive information about corticospinal excitability.

### Strengths and Limitations

The strength of this study is that it explored the relationship between TMS and neural oscillation from a network perspective using graph theory analysis. In the application of graph theory, a network is constructed by defining a series of nodes and connecting edges and the model can fit the pattern of brain activity, which makes it a great tool for brain functional segmentation and integration. In this study, graph theory was used to construct a brain network, and the relationship between neural oscillatory activities and TMS was explained from the perspective of the global network, which can help to explore the electrophysiological mechanism of TMS.

There are some limitations to this study. Firstly, we studied the association between EEG oscillation and TMS in non-dominant hemisphere, and the generalizability of the results to dominant hemisphere can be explored in future study. Besides, EEG were recorded before application of TMS in this study. Future study can record them simultaneously to provide stronger evidences. We chose the EEG spectrum and graph theory to analyze the relationship between EEG oscillation and cortical excitability because these are the classic metrics used to describe the characteristics of EEG. In future studies, other metrics such as entropy and complexity which quantifies the non-linear dynamic characteristics of EEG and reflect cortical functional state can be selected to explore the relationship between EEG oscillation and corticospinal excitability comprehensively.

## Conclusion

In conclusion, we found that the alpha2 power in the sensorimotor region showed an inverse correlation with RMT and AMT. And the global efficiency in the theta band was positively correlated with RMT. Additionally, the global efficiency in the alpha2 band was negatively correlated with RMT and AMT. It is crucial to understand the mechanisms of TMS from the perspective of neurophysiology. The findings of this study indicate that the effect of TMS on the cortex is dependent on the activity of local neurons and global network activity, which provides an important basis for uncovering the regulatory effect of TMS on neural electrical activity. The network analysis of EEG can provide a useful supplement for studying the brain response to TMS.

## Data Availability Statement

The raw data supporting the conclusion of this article will be made available by the authors, without undue reservation.

## Ethics Statement

The studies involving human participants were reviewed and approved by the Ethics Committee of Guangzhou First People’s Hospital. The patients/participants provided their written informed consent to participate in this study.

## Author Contributions

YL, GX, and TY: study design. QD, GC, MW, HCh, and HCa: data acquisition and processing. TL: statistical analysis. GC and MW: drafting of the manuscript. QD: critical revision of the manuscript. YJ and WL: technical assistance. All authors contributed to the article and approved the submitted version.

## Conflict of Interest

The authors declare that the research was conducted in the absence of any commercial or financial relationships that could be construed as a potential conflict of interest.

## Publisher’s Note

All claims expressed in this article are solely those of the authors and do not necessarily represent those of their affiliated organizations, or those of the publisher, the editors and the reviewers. Any product that may be evaluated in this article, or claim that may be made by its manufacturer, is not guaranteed or endorsed by the publisher.

## Footnotes

1
http://www.sccn.ucsd.edu/eeglab/

2
http://www.nitrc.org/projects/gretna/

3
http://www.R-project.org
